# The Heart in Space: Effects of Microgravity on Different Cell Types and Their Functions in the Cardiovascular System

**DOI:** 10.3390/biomedicines13102336

**Published:** 2025-09-24

**Authors:** Zenab Shahzad, Ramish H. Rafay, Niharika Bala, Yunus E. Dogan, Abdel A. Alli

**Affiliations:** 1 Department of Medicine, Division of Nephrology, Hypertension, and Renal Transplantation, University of Florida College of Medicine, Gainesville, FL 32610, USA; zenabshahzad@ufl.edu (Z.S.); rafay.r@ufl.edu (R.H.R.); niharikabala@ufl.edu (N.B.); 2 Department of Physiology and Aging, University of Florida College of Medicine, Gainesville, FL 32610, USA; yunusemredogan@yahoo.com; 3 Department of Pediatrics, Yahyalı State Hospital, 38500 Kayseri, Turkey

**Keywords:** microgravity, cardiovascular, endothelial cells, spaceflight

## Abstract

Space travel may have promising implications for innovative approaches in biomedical research. But there are potential challenges and health concerns associated with exposures from space travel that warrant the need for mechanistic studies to determine the effects on various organ systems including the cardiovascular system. Radiation exposure associated with space flight is known to adversely affect the heart and vascular system. However, less is known about the effects of microgravity on the cardiovascular system. Various functions of the cardiovascular system may be affected by microgravity. Studies have investigated changes in the cytoskeleton of various cell types in response to simulated microgravity. Other studies investigated the effect of microgravity on the permeability and migration of endothelial cells and myocardial atrophy associated with endothelial dysfunction. In addition, coagulation, vasoconstriction, blood volume, and cardiac dimensions were outcome measures of studies aimed at understanding the implications of microgravity on the cardiovascular system. This comprehensive review summarizes the effect of microgravity on various aspects of the cardiovascular system.

## 1. Introduction

With the advancement of science and technology, space flight has become feasible since Yuri Gagarin’s historic flight in 1961. However, these journeys have encountered some health concerns. Astronauts subjected to spaceflight frequently report substantial deterioration of bone microarchitecture and muscle mass, clinically categorized as osteoporosis and sarcopenia [1]. The microgravity environment of spaceflight weakens the immune system, making astronauts vulnerable to diseases [2]. Immune cells are sensitive to gravity changes, and microgravity affects the differentiation, activation, metabolism, and function of these cells through various mechanisms [2]. During space travel, endothelial cells exhibit impaired proliferation, heightened stress levels, and potential death [3]. There are numerous effects on the cardiovascular system in response to microgravity. Microgravity conditions have been shown to cause myocardial atrophy and dysfunction [4], affect cardiac remodeling [5], alter signaling in cardiomyocytes [6], and alter vasoconstrictor responses [7].

Researchers have explored the effects of both microgravity and hypergravity on endothelial cells, which form the inner lining of blood vessels and are crucial for vascular health [8]. Due to the high costs and logistical challenges of recreating true microgravity using parabolic flights or conducting experiments in space, many studies rely on ground-based simulation tools.

The aim of this review is to provide a comprehensive overview of the effects of microgravity on cardiovascular physiology, specifically endothelial function, myocardial structure, vascular tone, coagulation dynamics and cardiac remodeling, utilizing data from both in vivo and in vitro studies. We highlight the underlying mechanisms that are involved in these changes and aim to highlight possible countermeasures to mitigate microgravity-induced cardiovascular dysfunction.

## 2. Existing Methods to Simulate Microgravity

Several ground-based techniques have been developed to replicate microgravity conditions on Earth. These approaches employ diverse physical mechanisms, including rotation, random positioning, and magnetic levitation, to simulate the microgravity environment effectively. This chapter aims to examine the established methods used for microgravity simulation, focusing on their working principles, practical uses, and the benefits and drawbacks associated with each technique.

The clinostat is a widely used device for this purpose, as it continuously rotates cell cultures to randomize the direction of gravity, effectively mimicking microgravity conditions [9]. Researchers have also explored multiple methods to simulate microgravity beyond the clinostat, including devices like the Rotating Wall Vessel Bioreactor [10], Hindlimb Unloaded (HU) rodent model [11], and Magnetic Levitation systems [12]. While the Rotating Wall Vessel Bioreactor simulates low-gravity conditions by gently rotating cell cultures in a fluid-filled chamber, mimicking the free-fall environment of space, Magnetic Levitation offers a unique approach-using magnetic fields to counteract gravity’s pull, effectively suspending cells in a near-weightless state.

Though Magnetic Levitation has not yet been widely studied for its direct effects on endothelial cells, its potential is hinted at by earlier work on other cell types. For instance, experiments with mouse osteoblastic MC3T3-E1 cells demonstrated that levitation-based microgravity can influence gene expression [13], suggesting similar tools might one day help unravel how weightlessness impacts vascular cells. While this technique remains underexplored for endothelial studies, its success in bone cell research offers a promising pathway for future investigations into how blood vessel cells adapt-or struggle-under simulated space conditions.

By leveraging these alternative methods, scientists can broaden their toolkit for probing gravity’s role in cellular behavior, even as they work to refine existing approaches for more precise, Earth-based simulations [13]. Although many studies have examined isolated aspects of cardiovascular adaptation to microgravity, there is a lack of systematic reviews integrating these effects at the vascular and myocardial levels. Table 1 summarizes the published studies that employed microgravity or its surrogate simulations to investigate effects on cells and whole organisms.

## 3. Impact of Microgravity on the Actin Cytoskeleton and Permeability of Endothelial Cells

**Figure 1:**
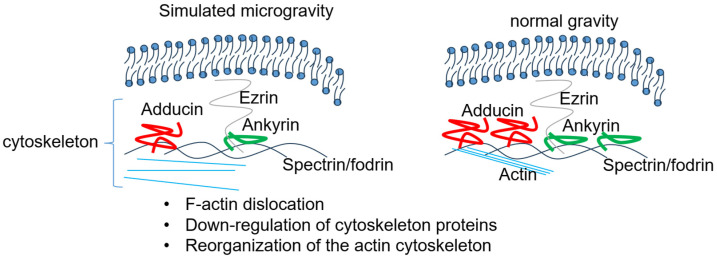
Schematic depicting effects of simulated microgravity on the actin cytoskeleton. The disassociation of f-actin [27], remodeling of the actin cytoskeleton [28], and downregulation of cytoskeleton proteins [29] by microgravity presumably alters the density and association of multiple actin associated proteins including ezrin, ankyrin, spectrin/fodrin, in specific cell types.

A study by Kang et al. showed rat aortic smooth muscle cells exposed to simulated microgravity had a disorganized cytoskeleton with fewer stress fibers, greater expression of the contractile phenotype marker smMHC, and less expression of the synthetic phenotype marker vimentin [23]. A study by Higashibata et al. showed that simulated microgravity causes disorganization of the actin cytoskeleton in bovine brain microvascular endothelial cells [24]. The results suggested that simulated microgravity using a 3D clinorotation apparatus downregulates LARG gene expression to reduce the activation of Rho, leading to disorganization of actin fibers [24]. In another study, Li et al., reported a decrease in the amount of F-actin and microtubules in EA.hy926 cells maintained under space microgravity conditions for 3 days [25]. In addition to the actin depolymerization, this group showed there was an accumulation in F-actin at the membrane in these cells in a time dependent manner. Baio et al. investigated the cytoskeletal dysregulation of neonatal and adult cardiovascular progenitor cells in response to the effects of spaceflight after being flown aboard SpaceX CRS-11 to the US National Lab [26]. The authors reported several cytoskeletal genes including NES, DES, VIM, LMNB2, and LMNA were all upregulated after spaceflight in neonatal but not adult cardiovascular progenitor cells. Shi et al. showed dissociation of F-actin and inactivation of RhoA in human umbilical vein endothelial cells maintained under simulated microgravity conditions. RhoA is a master regulator of actin cytoskeleton dynamics and plays an important role in polymerization of actin monomers [27]. A schematic depicting the effects of simulated microgravity on the actin cytoskeleton is provided in Figure 1. A list of some of the actin cytoskeleton associated proteins that are known to regulate cellular function is given in Table 2.

Spaceflight exposes astronauts to a unique and harsh environment that leads to profound physiological changes affecting multiple organ systems. The absence of gravity, exposure to cosmic radiation, confinement, and isolation all contribute to these effects, which can disrupt the normal functioning of cells in significant ways. Various systems including the pulmonary, musculoskeletal, immune and cardiovascular system can potentially be affected by microgravity. These adaptations, while sometimes necessary for survival in space, can have deleterious effects on health and function, both during missions and after returning to Earth. Perhaps the most important of these systems is the cardiovascular system as the endothelial cells are most sensitive to changes to conditions, inducing mechanical stress that alters the functioning of these cells [39].

**Figure 2:**
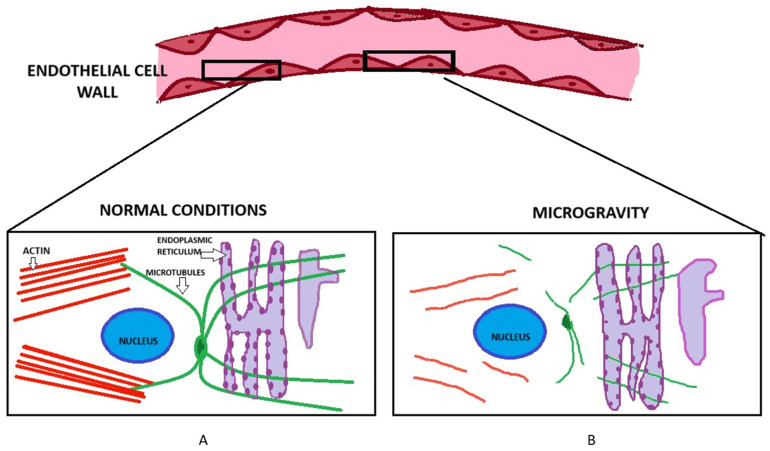
Changes in the structural integrity of endothelial cells from simulated microgravity. Box (**A**) shows a normal endothelial cell, under normal conditions and environment. The actin filaments (in red) and microtubules (green) are neatly arranged and maintain the structural support of the cells. Box (**B**) shows how the cytoskeleton of the endothelial cell changes after being in a simulated microgravity environment for 24 h. The actin filaments have decreased by about 65% which weakens the structural support of the cell. The microtubules are also visibly decreased and not maintaining its original shape [40]. This eventually causes increased permeability of cells.

Simulated microgravity has been known to increase endothelial cell permeability via multiple structural and molecular mechanisms. Exposing endothelial cells to simulated microgravity reduces the amount of actin filaments by approximately 65% and microtubules by 26%. This drastically reduces the overall ability of the cells to maintain the integrity of cytoskeleton [40]. Janmaleki et al. also showed in their study how endothelial cells lose up to 50% of their Young’s modulus within 24 h of exposure to simulated microgravity. This phenomenon diminishes their ability to maintain a barrier resistance and hence there is a decreased cell stiffness and viscosity. Similarly, exposure to microgravity also shows a reduction in the stiffness of cortical actin near the cells, resulting in loss or cortical rigidity which eventually compromises cell-to-cell adhesion and its barrier function [40]. The cumulative impact of these factors increases leakage between endothelial cells, shown by higher FITC-dextran permeability in microgravity-treated HUVEC layers [41]. Figure 2 shows changes in the structural integrity of endothelial cells in response to simulated microgravity.

## 4. Effect of Microgravity on Migration of Endothelial Cells

Simulated microgravity induces distinct functional responses across endothelial cell types, highlighting the complexity of cellular adaptation to low-gravity environments. In Human Umbilical Vein Endothelial Cells (HUVECs), exposure to microgravity conditions enhances migratory capacity, potentially through mechanosensitive pathways involving cytoskeletal reorganization or altered integrin signaling that facilitates directional movement [42]. Conversely, porcine aortic endothelial cells (PAECs) exhibit impaired migration under similar conditions, possibly due to dysregulation of adhesion molecule expression or disruptions in traction force generation required for cellular motility [8].

The antiproliferative effects of microgravity also demonstrate cell-type specificity. Murine microvascular endothelial cells show reduced proliferation rates when exposed to spaceflight analogs, which may correlate with metabolic reprogramming or changes in cell cycle progression regulators [43]. This inhibitory effect becomes more pronounced in HUVECs, where proliferation ceases entirely, suggesting either complete cell cycle arrest or activation of senescence pathways in this cell type. These divergent responses underscore the importance of cellular origin (vascular bed location) and species-specific factors in determining gravitational sensitivity [8,44].

Versari et al. designed a meticulous study to unravel how gravitational shifts influence the structural framework of human primary endothelial cells, particularly focusing on the actin cytoskeleton, a critical component for cell shape and movement [45]. To explore this, they employed a MidiCar centrifuge to expose cells to hypergravity, followed by microgravity conditions, creating a dynamic environment that mimics the gravitational transitions astronauts might experience during space missions [45].

For comparison, the team also investigated hypogravity effects on human umbilical vein endothelial cells (HUVECs) using two distinct simulation systems: the rotating wall vessel (RWV) and the random positioning machine (RPM). The RWV gently rotates cell cultures in a fluid-filled chamber, allowing cells to experience near-weightlessness as they remain suspended in constant free-fall motion. Meanwhile, the RPM randomizes gravity’s direction by continuously reorienting samples, effectively “averaging out” gravitational forces over time to simulate microgravity-like conditions. To assess cell migration, a key indicator of endothelial health, the researchers used an in vitro wound repair model, creating artificial gaps in the cell layer. They then observed how HUVECs cultured in the RPM migrated to close these gaps. Using a light microscope paired with a grid, they meticulously counted the number of cells moving into the wound area, providing precise data to quantify migration rates under simulated hypogravity. This approach allowed them to connect gravitational stress with cytoskeletal behavior, offering insights into how endothelial cells adapt—or struggle—when gravity’s pull is altered. By leveraging these tools, the investigators bridged hypergravity and hypogravity studies, highlighting how endothelial cells dynamically reorganize their internal architecture in response to gravitational changes, with potential implications for astronaut health and vascular research [45]. Western blotting was used to identify specific protein changes linked to mechanical stress, while immunofluorescence imaging revealed how the cells’ structural components, including the actin cytoskeleton, physically reorganize under these forces. HUVECs treated with Hepatocyte Growth Factor (HGF) showed a dramatic boost in migration speed [46]. These cells moved more aggressively to close gaps in the wound repair model, which could suggest that microgravity and HGF may work together to “switch on” pro-migration pathways. This synergy hints at how space-like conditions could amplify growth factor signaling, potentially reshaping how we understand wound healing or vascular repair in low-gravity environments. Shi et al. also performed an experiment to determine how after 24 h of simulated microgravity via clinostat, N-nitro-L-arginine methyl ester hydrochloride (L-NAME), an endothelial nitric oxide synthase (eNOS) inhibitor, affects the migration of endothelial cells [41]. Nitric oxide (NO) generated by endothelial nitric oxide synthase (eNOS) plays a role in controlling blood vessel tone, influencing the restructuring of blood vessels, and supporting the formation of new blood vessels (angiogenesis) [47,48,49]. L-NAME was added to the initial culture media and the results were seen through a tube formation assay [41]. It was seen that L-NAME significantly blocks HUVEC-C tube formation under simulation of microgravity via the 2D clinostat. Tube formation in vitro requires both attachment of cell to extracellular matrix and cell migration. Cell motility was evaluated using a scratch assay, where a uniform scratch was made in a confluent layer of cells to create a gap, or “wound.” This allowed the investigators to observe how the cells migrated to close the gap over time. To specifically assess how endothelial cells moved under simulated microgravity conditions, a wound healing assay was performed. By monitoring how quickly and effectively the cells migrated to fill the scratch, the effects of microgravity on their migratory behavior could be identified. By inhibiting eNOS activity, L-NAME counteracted the pro-angiogenic effects of simulated microgravity on HUVEC-C tube formation therefore also decreasing endothelial cell migration [41].

## 5. Effect of Microgravity on Maturation of Cardiomyocytes and Progenitor Stem Cells

A study by Jha et al. showed cardiac progenitors exposed to simulated microgravity promoted the production of enriched cardiomyocytes [50]. In a follow-up study by Forghani et al. the group investigated the maturation of cardiomyocytes derived from human induced pluripotent stem cells and found that microgravity increased mitochondrial respiration, improved calcium transient parameters, and improved structural properties [51]. In a different study, Lopez Garzon et al. showed cardiomyocytes derived from human pluripotent stem cells that were maintained under microgravity conditions showed enhanced mitochondrial biogenesis [52].

Microgravity has significant effects on various stem cell types, influencing their proliferation, differentiation, and gene expression profiles. Microgravity inhibits the proliferation of hematopoietic stem cells by blocking the cell cycle at the G/S transition and downregulating genes related to cell proliferation while upregulating genes related to cell death. This is mediated through pathways such as the Kit-Ras/cAMP-CREB pathway. Additionally, microgravity causes dynamic alterations in blood lineage cells, with reductions in NK cells, B cells, and erythrocyte precursors, and an increase in T cells, neutrophils, and hematopoietic stem cells [53,54].

Microgravity induces oxidative stress in mesenchymal stem cells, leading to premature senescence and a loss of stemness and proliferation capability. This is associated with the modulation of stress and stemness-related genes, as well as cell proliferation regulators. Furthermore, microgravity impacts the gene expression of mesenchymal stem cells, affecting pathways related to cell division, chromosomal segregation, and extracellular matrix structure [55,56].

Exposure to microgravity results in enhanced proliferation and abnormal cell division in neural stem cells, including incomplete cell division and multi-daughter cell division. These changes have implications for understanding the mechanisms leading to neurological disorders and potential carcinogenic susceptibility [57]. Simulated microgravity decreases the proliferation and differentiation of bone marrow stem cells, blocking the cell cycle in G2/M and enhancing apoptosis. This is linked to decreased expression of SATB2, a key regulator of osteoblast differentiation [58].

Cerebral venous drainage occurs mainly through the internal jugular veins (IJV) in the supine position. In the upright posture, the IJVs collapse due to intraluminal pressure being less than the atmospheric pressure. Most cerebral outflow in this state occurs through the vertebral veins and vertebral plexus [59]. However pressure in the IJV is observed to be elevated during short periods of weightlessness in parabolic flights [60]. Marshall-Goebel et al. investigated jugular venous flow dynamics during acute weightlessness using ultrasound imaging [60]. They found that the IJV cross-sectional area increased significantly during spaceflight, indicating venous distension. Additionally, stagnant flow and retrograde flow were observed in the IJV of 6 out of 11 participants during weightlessness. Another finding was the presence of an occlusive and partially occlusive thrombi in the IJV of 2 crew members [61]. Another study by the same author reported bilateral distension of IJVs during spaceflight with an increased risk of flow stasis in the left IJV during acute weightlessness [62].

**Figure 3:**
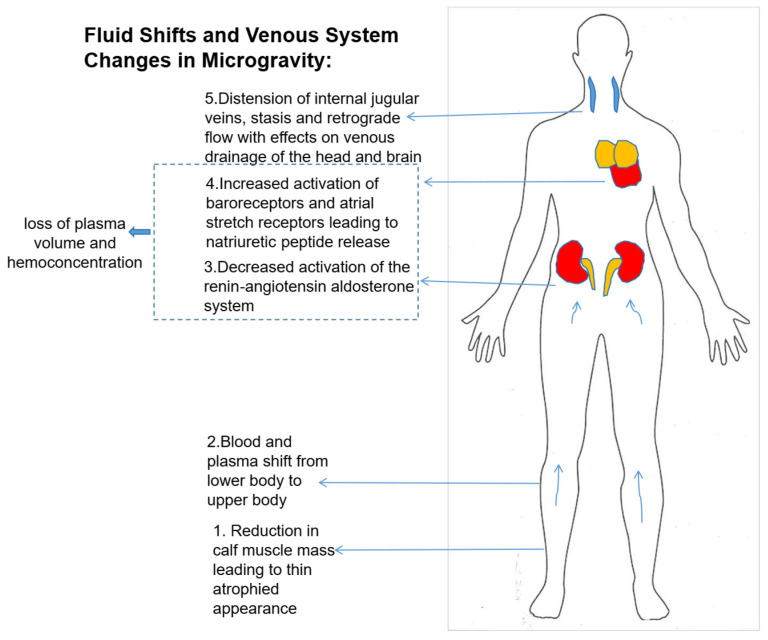
Effects of microgravity on fluid-redistribution throughout the body and resulting changes. The arrows show the direction in which blood and plasma shifts under simulated microgravity.

A study by Cohen et al. also demonstrated region specific differences in the ability of the IJV to distend [62]. Their results show that the caudal region of the IJV can accommodate significantly more distension than the cranial region. This leads to irregular flow patterns and can contribute to adverse flow profiles leading to increased risk of thromboembolism [63]. Lee et al. characterized vascular changes during partial gravity exposure. They observed progressive increases in IJV cross-sectional area and shifts in flow patterns from continuous forward flow to pulsatile flow as gravitational levels decreased, suggesting that partial gravity levels greater than 0.50-G may be required to reduce weightlessness-induced fluid shifts [64]. Figure 3 summarizes the effects of microgravity on fluid-redistribution throughout the body. A decrease in muscle mass in the lower extremities of the body causes blood to shift from the lower limbs upwards. Next, downregulation of the renin-angiotensin system occurs along with baroreceptor activation leading to the release of natriuretic peptides. Another change is the widening of the internal jugular veins leading to disruption in the venous drainage of the head.

## 6. Effect of Microgravity on Coagulation

Virchow’s triad is a conceptual framework with 3 components which explain and increase the risk of thromboembolism and are also involved in hemostasis. These 3 components include vascular endothelial injury, sluggish or turbulent blood flow and hypercoagulability. Endothelial injury exposes underlying vascular tissue and provides a prothrombotic surface favorable for platelet adhesion and activation of the coagulation cascade. Turbulent blood flow can cause platelets to activate by exposure to abnormal shear stresses and stagnant blood concentrates cellular and soluble components of coagulation, both increasing the risk of clot formation [65].

Space travel affects the cardiovascular system by disrupting normal fluid distribution. In microgravity, fluids shift toward the head due to the loss of gravity-driven pressure gradients and decreased thoracic pressure [60]. This leads to swollen facial features and distended neck veins [66]. The jugular vein’s pressure is markedly higher in the preflight 1.00-G_z_ supine compared to the seated 0.50 G_z_ position, with its cross-sectional area increasing almost threefold [64]. Similarly, the portal vein may significantly expand during prolonged weightlessness [67]. Blood flow through these enlarged vessels also changes in space. Ultrasound imaging has shown stagnation, increased turbulence, and even reversed flow in astronauts’ internal jugular veins while in spaceflight [61]. Indeed, spaceflights and microgravity seem to be associated with an increased risk of coagulation and thrombotic events. Observational studies have documented thrombotic events in astronauts, such as the internal jugular vein thrombosis reported during spaceflight, highlighting the potential thrombotic risks associated with microgravity [68,69].

Microgravity and spaceflight have a significant impact on the proteins associated with coagulation. A study to check the effects of long duration missions to the ISS on 125 blood proteins sampled from 18 cosmonauts found that proteins involved in coagulation (factor XI, fibrinogen, fibrinopeptide A, and the serpin plasminogen activator inhibitor-3) and the acute phase reactant haptoglobin were increased on the first day of spaceflight [70]. Increased fibrinogen was also reported by 13.2% in astronauts on shorter duration missions [70]. Experimental studies, including ground-based analogs like head-down bed rest and parabolic flights, have been used to simulate the effects of microgravity. Studies have shown changes in venous flow dynamics [62], increased fibrinogen levels, and the presence of thrombin generation markers, indicating a hypercoagulable state [71,72].

Research on platelet functions has shown different results. A study using the dry immersion (DI) model, in which healthy male adults were exposed to simulated microgravity, demonstrated that a 3-day DI exposure resulted in a significant increase in platelet count, platelet crit, platelet adhesion, aggregation, and a modest elevation of the platelet reactivity index (PRI). This model induced a prothrombotic platelet phenotype, with changes in the miRNA signature and circulating plasma protein biomarkers [73]. Another study in which mice underwent tail suspension and unloaded hindlimb found that simulated microgravity inhibits platelet functions, increases tail-bleeding time, decreased aggregation induced by ristocetin or collagen and adhesion to von Willebrand factor (VWF). This inhibition was associated with decreased surface expression of glycoprotein (GP) Ibalpha and its association with the cytoskeleton [74].

## 7. Simulated Microgravity’s Effects on Vasoconstriction

Vasoconstriction plays a crucial role in maintaining homeostasis in a healthy individual. It is primarily mediated by the sympathetic nervous system, which releases norepinephrine and other transmitters such as neuropeptide Y to induce the constriction of blood vessels [75]. This process reduces blood flow to the skin and extremities, thereby conserving heat and maintaining core body temperature during cold exposure [75,76]. Additionally, vasoconstriction helps regulate blood pressure by increasing vascular resistance, which is essential for ensuring adequate perfusion of vital organs. The delicate balance between vasoconstriction and vasodilation is crucial for maintaining overall physiological function and allows the body to respond to various internal and external changes. Microgravity has been shown to significantly impact vasoconstriction, particularly in mesenteric arteries and veins. Studies have demonstrated that both spaceflight and simulated microgravity conditions lead to diminished vasoconstrictor responses.

A study by Behnke BJ et al., investigated orthostatic hypotension experienced by astronauts due to reduced peripheral vascular resistance following spaceflight [77]. A mouse model was used to test the hypothesis that mesenteric arteries and veins exhibit diminished vasoconstrictor responses after spaceflight. In vitro studies on the mesenteric arteries showed that spaceflight decreased arterial ryanodine receptor-3 mRNA levels. In veins, norepinephrine-induced constriction was diminished after flight. The impaired vasoconstriction following spaceflight was attributed to ryanodine receptor-mediated intracellular Ca^2+^ release. These vascular changes could compromise arterial pressure maintenance during orthostatic stress in astronauts [77]. In another study by Stabley JN et al. that addressed cardiovascular adaptations to microgravity, the hypothesis that vasoconstrictor responses mediated through adrenergic receptors norepinephrine (NE), voltage-gated Ca^2+^ channels (KCl), and stretch-activated (myogenic) mechanisms would be diminished following spaceflight was tested using a similar space model and in vitro studies on isolated gastrocnemius muscle feed arteries were performed [78]. This group found that spaceflight reduced vasoconstrictor responses to potassium chloride (KCl) and NE, while myogenic vasoconstriction remained unaffected. The diminished vasoconstrictor responses were associated with lower ryanodine receptor-2 (RyR-2) and ryanodine receptor-3 (RyR-3) mRNA expression, with no difference in sarcoplasmic/endoplasmic Ca^2+^ ATPase 2 mRNA expression. Vessel wall thickness and maximal intraluminal diameter were unaffected by spaceflight. The study concluded that spaceflight impairs vasoconstrictor responsiveness in skeletal muscle resistance arteries due to a deficit in intracellular calcium release via RyR-2 and RyR-3 in smooth muscle cells. The data suggested that impairment contributes to lower peripheral vascular resistance and reduced tolerance to orthostatic stress in humans after spaceflight [78]. The study provides robust evidence that spaceflight impairs vasoconstrictor responsiveness in skeletal muscle resistance arteries, primarily due to a deficit in intracellular calcium release via RyR-2 and RyR-3. This finding is significant as it helps explain the lower peripheral vascular resistance and reduced tolerance to orthostatic stress observed in humans after spaceflight.

A study by Sangha et al. investigated the effects of simulated microgravity on vascular responsiveness, particularly focusing on the role of endothelial-derived vasoconstrictor prostaglandins [79]. The researchers simulated microgravity in rats using hindlimb unweighting (HU) for 20 days. Carotid arteries were isolated from both control and HU-treated rats, and vascular rings were mounted in tissue baths to measure isometric contraction. Cyclooxygenase inhibitors (indomethacin and ibuprofen) and a selective thromboxane A2 prostanoid receptor antagonist (SQ-29548) were used to assess their effects on norepinephrine (NE) induced contraction. The study found that these inhibitors significantly reduced NE-induced contraction in HU vessels but had no effect on control vessels. Removal of the endothelium negated the effect of indomethacin on NE-induced contraction in HU vessels. Additionally, the presence of indomethacin and a nitric oxide synthase inhibitor (N(G)-L-nitro-arginine methyl ester) increased NE induced contraction in HU vessels to levels comparable to control vessels. The findings indicate that simulated microgravity induced two opposing endothelial changes including an increase in nitric oxide activity, which causes vascular hypo responsiveness to NE, and an increase in activity of the vasoconstrictor prostaglandin, which attenuates the vasodilating effect of nitric oxide. This dual modulation suggests a complex endothelial adaptation to simulated microgravity, highlighting the role of cyclooxygenase products in vascular function under these conditions [79]. The study is robust in its methodology, using well-established techniques such as hindlimb unweighting to simulate microgravity and employing pharmacological agents to dissect the roles of different endothelial factors. The findings are significant as they highlight the intricate balance between vasodilatory and vasoconstrictive mechanisms in response to altered gravitational forces. The use of both cyclooxygenase inhibitors and a selective thromboxane A2 receptor antagonist strengthen the evidence for the involvement of cyclooxygenase products in the observed vascular changes. However, the studies above are limited by their use of an animal model, which may not fully replicate the human physiological response to microgravity. Additionally, the focus on carotid arteries or on the gastrocnemius muscle feed arteries may not capture the full spectrum of vascular adaptations occurring in other vascular beds. Future studies should aim to validate these findings in human subjects and explore the implications for other vascular regions.

**Figure 4:**
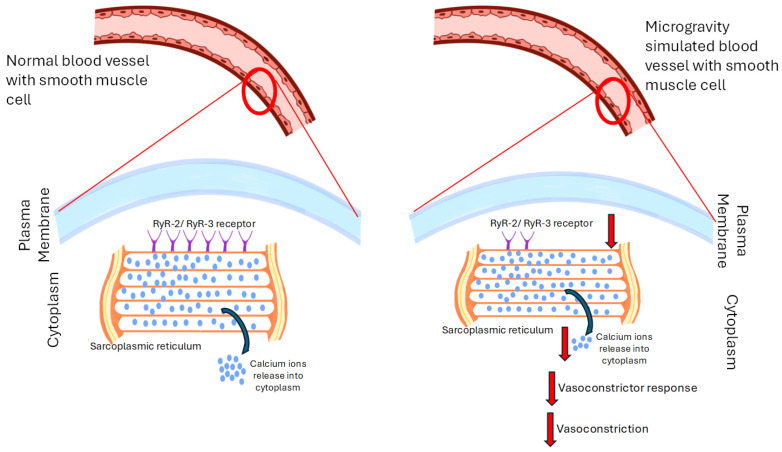
Schematic on Effects of Microgravity on Vasoconstriction. This figure shows that vascular smooth muscle cells exposed to microgravity have decreased expression of the RyR-2/RyR-3 receptors being expressed on the sarcoplasmic reticulum there by alleviating the intracellular release of calcium ions into the cytoplasm which leads to reduced responsiveness to vasoconstrictors.

Collectively, these studies suggest that spaceflight and simulated microgravity significantly impair vasoconstrictor responses in arteries and veins, primarily due to reduced peripheral vascular resistance and altered calcium signaling in vascular smooth muscle cells. Studies show decreased expression of ryanodine receptor-2 and -3, leading to diminished intracellular Ca^2+^ release and reduced responsiveness to vasoconstrictors like norepinephrine and potassium chloride. This impairment compromises arterial pressure regulation and increases the risk of orthostatic intolerance upon return to gravity. The findings, observed in both animal models and in vitro studies, highlight that spaceflight-induced vascular dysfunction is linked to molecular changes affecting smooth muscle contraction and vascular tone. The effects of microgravity on vasoconstriction are summarized in Figure 4.

Consistent with this study, another study by White et al., investigated the mechanisms underlying vascular hyporesponsivity following acute simulated microgravity [14]. The study utilized a 3-day hindlimb unloaded (HU) rat model to simulate microgravity conditions. Results showed that vasoconstrictor responses to NE were significantly depressed in HU rats, but endothelial removal or inhibition of the NO/cGMP pathway restored vascular contractility. Increased NOS activity and NO products were observed, along with altered phosphorylation states of endothelial nitric oxide synthase (NOS-3). Key conclusions indicate that the reduced vasoreactivity in acute HU rats is due to an upregulation of the endothelium-dependent NO/cGMP pathway through NOS-3 phosphorylation, highlighting the role of endothelial mechanisms in microgravity-induced vascular changes [14].

In contrast to the above studies, a study has shown that simulation microgravity can enhance vasoconstrictor responsiveness. For instance, Zhang et al., have used a 4-week tail-suspended hindlimb unloading model to simulate microgravity [7]. The methods involved isolating basilar arterial rings from both tail-suspended (TS) and control rats and examining their responsiveness to various vasoconstrictors (KCl), arginine vasopressin, and 5-hydroxytryptamine) and vasodilators (ACh, thrombin, adenosine, and sodium nitroprusside). The results showed that the maximal isometric contractile responsiveness to vasoconstrictors was significantly enhanced in basilar arterial rings from TS rats compared to control rats, while vasodilatory responsiveness remained unchanged. The group concluded that the phenomenon of enhanced vasoconstriction in TS rats is due to an impairment of endothelium-dependent mechanisms, suggesting that endothelium-derived hyperpolarizing factors play a role in modulating the contractile responsiveness of rat basilar arteries to 5-HT [7]. Additionally, Lin et al. aimed to investigate the impact of simulated microgravity on vascular structure and function, specifically focusing on middle cerebral arteries (MCAs) and mesenteric small arteries (MSAs) [80]. The researchers employed a 28-day tail suspension model to simulate microgravity and examined the effects of daily 1 h dorsoventral (-Gx) gravitation as a countermeasure. The results revealed that simulated microgravity induced hypertrophy and enhanced vasoconstrictor reactivity in MCAs, while causing atrophy and decreased vasoconstrictor responses in MSAs. Daily -Gx gravitation effectively prevented these structural changes and functional decrements in MSAs but did not mitigate the increased myogenic tone and vasoreactivity in MCAs. The study concluded that small resistance arteries exhibit distinct adaptive features to microgravity, and daily gravitational loading can serve as a countermeasure to prevent adverse vascular changes [80]. In summary, the effect of microgravity on vasoconstriction might be different for acute and chronic phases of exposure, and apart from the time of exposure, the effect of microgravity might be different between various tissues in our body.

## 8. Effect of Microgravity on Blood Volume and Hematologic Changes

A few studies have shown that microgravity or spaceflight can induce change in blood volume and cause anemic conditions. Lampe et al. have performed a study which involved six healthy male subjects whose blood samples were collected at various intervals before, during, and after head-down tilt (HDT) exposure [81]. The group showed reduced red cell deformability which indicated diminished quality of the red blood cells and increased red cell aggregation, attributed to elevated hematocrit levels and decreased plasma protein concentrations. The study concluded that HDT conditions mimic spaceflight-induced anemia, highlighting the role of diminished physical activity and increased hemoglobin concentration in altering blood fluidity [81]. Another study by Wang et al., explored the molecular mechanisms by which microgravity affects hematopoietic stem cells (HSPCs) [54]. Using mouse models, the study maintained HSPCs under spaceflight and simulated microgravity conditions and then measured cell proliferation and gene expression. Results indicated a significant decrease in HSPC numbers due to cell cycle arrest at the G/S transition, with down-regulation of proliferation-related genes and up-regulation of cell death-related genes. The study identified the Kit-Ras/cAMP-CREB pathway as a key regulator affected by microgravity, providing insights into the intracellular mechanisms inhibiting HSPC proliferation [54]. Cao et al. examined the impact of microgravity on blood lineage cells and hematopoietic stem cells (HSCs) [53]. Peripheral blood samples from astronauts and a mouse model using hindlimb unloading were analyzed. Results showed significant changes in cell populations, including reductions in NK cells, B cells, and erythrocyte precursors, and increases in T cells, neutrophils, and HSCs. Recovery rates varied among cell types, with T cells recovering faster than others. The study concluded that microgravity disrupts immune system homeostasis and impairs HSC function, although these changes are reversible [53]. In conclusion, these studies collectively highlight that microgravity induces significant hematologic changes, including impaired blood fluidity, inhibited HSC proliferation, and dynamic alterations in blood lineage cells. These findings underscore the need for targeted countermeasures to mitigate the adverse effects of microgravity on the hematopoietic system during extended space missions.

## 9. Myocardial Atrophy and Endothelial Dysfunction

**Figure 5:**
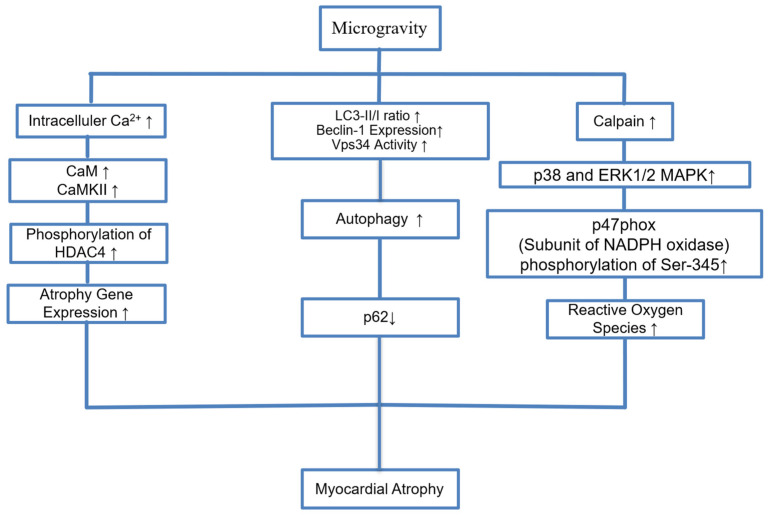
Pathophysiological Mechanisms of Myocardial Atrophy in response to microgravity. Microgravity conditions lead to an increase in intracellular calcium, Vsp34 activity, and calpain activity via distinct mechanisms. The ↑ and ↓ arrows represent upregulation and downregulation respectively.

Initial studies using chest X-rays by NASA showed that 80% of Apollo astronauts had a reduced cardiothoracic ratio [82]. Echocardiography later demonstrated decreased LV end-diastolic dimension (LVEDD), stroke volume (SV), and mass upon return to Earth. These findings were confirmed by cardiovascular magnetic resonance in astronauts and bed rest patients, suggesting that microgravity causes morphological atrophy of the heart [83]. Animal studies have also shown myocardial atrophy in mice in microgravity models [15]. Liang et al. found that calpain was increased in mice in a microgravity model and calpain increases phosphorylation of p47phox protein, a subunit of NADPH oxidase, at the Ser-345 position via the p38 and ERK1/2 MAPK signaling pathways [4]. They found that when calpain is genetically silenced or suppressed with chemical inhibitors, these detrimental effects are significantly reduced [4]. Another proposed mechanism for atrophy is autophagy [16]. Autophagy-related markers (LC3-II/I, Beclin-1, Vps34) and autophagy flux (decreased p62) were found to be increased by the tail suspension-induced microgravity model. This suggests that TS may enhance autophagic activity. The lysosome inhibitor chloroquine (CQ) has been shown to improve TS-induced cardiac dysfunction. There was no difference in apoptosis-related markers [16]. Microgravity has also been shown to induce cardiac atrophy and myofibrillar replacement in which the Ca^2+^-CaMKII-HDAC4 axis plays a central role. Increased intracellular Ca^2+^ activates CaM and CaMKII, leading to HDAC4 phosphorylation [82]. This mechanism is illustrated in Figure 5.

In addition to mechanisms that cause atrophy, such as oxygen radicals and autophagy in endothelial injury, microgravity also triggers a series of molecular mechanisms that lead to vascular dysfunction. Endoplasmic reticulum (ER) stress causes disruption of protein homeostasis and mitochondrial Ca^2+^ overload, increasing fission and triggering apoptosis. In this process, the PTEN-PINK1-Parkin pathway is activated, accelerating mitochondrial degradation through mitophagy [17]. In this case, it impairs cell permeability. At the same time, microgravity increases the expression of adhesion molecules such as ICAM-1, VCAM-1, E-selectin and VE-cadherin, exacerbating leukocyte adhesion and vascular inflammation [82]. Activation of the NF-κB and NLRP3 inflammatory pathways increases the release of pro-inflammatory cytokines such as TNF-α, IL-6 and IL-1β, leading to chronic inflammation. In addition, nitric oxide (NO) imbalance develops. Alterations in eNOS/iNOS expression in arteries result in hypotension due to excessive NO production or vascular stiffness due to NO depletion. Dysregulation of eNOS phosphorylation (increased Ser1177, decreased Ser495) contributes to uncontrolled NO production, exacerbating the hemodynamic imbalance [14,18]. All these pathophysiological processes lead to endothelial dysfunction. Certain recommendations can protect against these adverse effects. Exercise is the most effective way to counter cardiovascular deconditioning in space. Resistance and aerobic exercises in ISS prevented changes in cardiac mass and volume. In addition to exercise, artificial gravity (e.g., lower body negative pressure, short-arm centrifugation) is also promising for cardiovascular protection [19,84]. Time-restricted feeding (TRF) is effective in reducing microgravity-induced cardiac dysfunction. Through the activation of the FGF21 pathway, it improves the heart’s glucose utilization and contractile function [20]. In addition, plant components such as Panax quinquefolium saponin show protective effects against cardiac remodeling and vascular dysfunction [21].

Although not yet standardized, pharmacological approaches targeting certain biochemical pathways have shown promise. For example, treatment with miR-199a-3p has shown potential in reducing cardiac remodeling [22]. Furthermore, agents targeted at reducing endoplasmic reticulum stress and associated inflammatory responses have been found useful in reducing endothelial dysfunction [18].

These findings and proposed therapies collectively emphasize that microgravity causes cardiac atrophy through converging but distinct molecular mechanisms such as oxidative stress, calcium dysregulation and autophagy. Although multiple pathways such as the CaMKII-HDAC4 axis, calpain activation and mitophagy have been implicated, studies looking at them all together are lacking. Future research goals should focus on taking a holistic view of the effects on cardiac and vascular tissue, prioritizing therapeutic targets and their adverse effects as well.

## 10. Microgravity and Changes in Cardiac Dimensions

Prolonged exposure to microgravity, such as in spaceflight, leads to a reduction in ventricular wall stress in the heart. This is due to the absence of gravitational loading [19]. Changes in cardiac work, including stroke volume and left ventricular end-diastolic volume accompanied with a loss of gravitational stress have been well described during simulated microgravity (6° head-down tilt) on Earth [85]. This leads to a decrease in cardiac mass and volume, a pathological change known as cardiac atrophy. However, exercise countermeasures used on the International Space Station (ISS) have been shown to mitigate these reductions in cardiac mass and volume, despite overall reductions in total cardiac work [19].

Microgravity has also shown to alter the geometry of the heart. Videbaek and Norsk demonstrated that short periods of microgravity during parabolic flights lead to an increase in left atrial diameter due to changes in transmural central venous pressure [86]. A study by Perhonen et al. used MRI to quantify LV mass in astronauts and showed that short duration spaceflights of 10 days led to a decreased left ventricular mass [83].

On a molecular level, many of the atrophic changes in heart in microgravity environments are mediated through proteases for example, Calpain, a calcium dependent protease has been shown to promote myocardial abnormalities in microgravity via the ERK1/2 and p38 MAPK pathways [4]. Spaceflight has also shown to alter the expression of key molecules involved in oxidative stress. A study by Kumar et al. on mice flown aboard the STS-131 mission on the Space Shuttle Discovery, showed altered cardiac expression of genes related to cell cycle/growth (notably Cdkn1a/p21, Cdk1, and Myc), inflammation (notably Tnf) and oxidative stress (notably Nfe2l2, Nox1, Ptgs2) [87]. The study showed an increased oxidative stress and impaired defense against reactive oxygen species in heart tissue [87].

## 11. Several Countermeasures Are Being Investigated to Offset the Detrimental Effects of Microgravity on Human Physiology

The evidence to date indicates that standalone nutritional interventions have limited efficacy in preventing cardiovascular deconditioning associated with microgravity. Most nutritional countermeasures such as increased protein intake, amino acid supplementation, and antioxidant cofactors did not meaningfully improve cardiovascular outcomes during simulated microgravity or bed rest [88]. The technique of time-restricted feeding (TRF) has shown promise in animal models. In a rat hindlimb unloading model, TRF (food access limited to 8 h per day) preserved cardiac function and improved cardiac glucose utilization [20]. However, these findings have not yet been validated in human spaceflight or bed rest studies. One paper investigated the effect of bed rest (an analog of microgravity) and diet on vasodilatory ability of blood vessels. It showed that short-term bed rest impaired endothelium-dependent arterial relaxation in humans. A hypoenergetic, low-fat diet decreased serum lipids, improves endothelium-dependent and -independent relaxation. This might counteract the negative impact of simulated microgravity on endothelial function and have positive significance for space travel [89].

Current research on reducing the cardiovascular effects of microgravity through novel drug therapies is in early stages with no drug regimen currently established as standard of care for cardiovascular protection in microgravity. Apart from the use of midodrine [90] to treat post spaceflight orthostatic hypotension, there has been little development in pharmacological therapies for cardiovascular effects. One path that is being explored is drug repurposing. Microgravity induces dysregulation of several genes. These can be targets for therapeutic interventions [91]. An agent that acts as an agonist of Piezo1, a mechanosensitive calcium (Ca^2+^) ion channel has already been designed and could be used to treat bone resorption associated with microgravity [92].

Despite all these developments, structured exercise regimens, including both aerobic and resistance modalities, remain the most effective countermeasures to cardiovascular deconditioning in microgravity environments. Recent data demonstrate that these exercise countermeasures are effective in offsetting reductions in cardiac mass and volume during prolonged spaceflight, despite an overall reduction in total cardiac work. Specifically, left ventricular mass and function are largely preserved in astronauts who adhere to these regimens [19]. Ground-based analogs, such as head-down bed rest (HDBR), have been used to simulate microgravity. Studies show that high-intensity physical exercise (e.g., jump training) during HDBR can attenuate cardiovascular deconditioning, as evidenced by preserved stroke volume and VO2 max, and reduced decline in cardiac kinetic energy compared to controls. Combined resistance and aerobic exercise during bed rest also helps maintain neuromuscular and cardiovascular function, reducing the extent of postural and functional deficits observed after simulated microgravity [93].

## 12. Future Perspectives

Future studies should concentrate on integrated techniques combining molecular, cellular, and hemodynamic data to produce a consistent model of cardiovascular adaptation and damage in space environments given the complexity of cardiovascular responses to microgravity. Key regulatory nodes engaged in cytoskeletal dysregulation, oxidative stress and endothelial dysfunction can be found using high-throughput methods such as transcriptomics, proteomics, and single-cell sequencing. Furthermore, investigating the chronological development of these changes from acute to chronic exposure may help identify important opportunities for intervention. Furthermore, bridging the gap between mouse models and human physiology may be achieved by performing functional investigations employing organ-chip technologies and human-derived stem cells. In the end, exact molecular targets could help to create efficient countermeasures to preserve cardiovascular health throughout long-term spaceflights. As space exploration advances toward longer missions and potential colonization, deeper insight into the mechanisms underlying microgravity-induced vascular dysfunction is essential. Future studies should investigate region-specific and time-dependent variations in vasoconstrictive responses, with a focus on intracellular calcium dynamics and endothelial signaling. Additionally, understanding hematologic alterations at the molecular level, especially the recovery kinetics of hematopoietic and immune cell populations, will be crucial for developing effective countermeasures. Integrating omics-based profiling with in vivo simulation models may unlock novel therapeutic targets to mitigate cardiovascular and hematologic risks in spaceflight.

Future research should focus on leveraging advanced model systems such as organ-on-a-chip technologies and human induced pluripotent stem cell-derived endothelial cells to better understand the molecular mechanisms underlying microgravity-induced endothelial dysfunction and to develop targeted countermeasures for cardiovascular protection during long-duration space missions. Additionally, expanding studies to explore the cross-talk between endothelial cells and other cell types in three-dimensional culture platforms and real microgravity conditions will be critical for unraveling the complex tissue-level responses and improving strategies for tissue repair and vascular health in space and on Earth. Researchers use various ground-based tools, like clinostats, rotating bioreactors, and magnetic levitation systems, to simulate microgravity and study its effects on endothelial cells, which are vital for blood vessel health. While each method has limitations, ongoing improvements and the use of advanced technologies will help scientists better understand how gravity changes impact vascular cells and develop strategies to protect cardiovascular health during space missions and on Earth. Simulated microgravity affects different endothelial cell types in unique ways, influencing their migration, proliferation, and structural organization through changes in cytoskeletal dynamics and signaling pathways like nitric oxide. Understanding these cell-specific responses is crucial for developing strategies to protect vascular health during space missions and requires further research using both advanced laboratory models and real spaceflight experiments.

## 13. Conclusions

Published studies have shown that the integrity of the cytoskeletal of various cell types is disrupted by the gravitational differences between Earth and space and this leads to changes in cell morphology, intracellular signaling, and cellular function. Microgravity affects several molecular mechanisms leading to morphological atrophy and vascular dysfunction in the heart. Pathophysiological pathways such as oxidative stress, auto-compensation, calcium signaling pathways, inflammation and mitochondrial dysfunction are involved in this process. Microgravity has also been shown to have deleterious effects on progenitor stem cells with dysregulation of the cell cycle. This is shown to happen through modulation of genes associated with cell cycle and cell death and through oxidative stress. The cephalad fluid shift in a microgravity environment leads to distension of the IJV with stagnation in blood flow and even retrograde flow. This, combined with endothelial injury and a hypercoagulable profile, increases the risk of thrombotic events. Spaceflight and simulated microgravity profoundly disrupt vascular and hematologic homeostasis. Microgravity impairs vasoconstrictor responses in arteries and veins, mainly due to altered calcium signaling and decreased expression of ryanodine receptors, resulting in reduced peripheral vascular resistance and increased risk of orthostatic intolerance upon return to gravity. Endothelial adaptations under microgravity involve both increased nitric oxide activity and changes in prostaglandin signaling, leading to complex and sometimes opposing effects on vascular tone. Additionally, microgravity induces significant hematologic changes, including reduced red blood cell deformability, anemia-like conditions, and impaired hematopoietic stem cell proliferation through molecular pathways such as Kit-Ras/cAMP-CREB. These alterations also affect immune cell populations, disrupting immune homeostasis, though some changes are reversible after returning to normal gravity. Although published studies have been conducted to investigate the effects of simulated microgravity on various aspects of the cardiovascular system, additional studies are needed to better understand the pathophysiology at the molecular level.

## Figures and Tables

**Table 1:** Summary of the models used and their main findings in simulated microgravity in various studies described in this review.

Ref. No	Study	Model Used	Main Findings	Key Molecular Targets
[4]	Liang et al., 2020	Tail-suspension was conducted to simulate microgravity in mice (WT and cardiomyocyte-specific Capns1 knockout)	Microgravity induced myocardial apoptosis, mitochondrial damage, and cardiac dysfunction; calpain activation plays a central role via MAPK pathways	Calpain, NADPH oxidase (p47phox, p67phox, Rac1), ERK1/2, p38 MAPK
[14]	White et al., 2010	Rat hindlimb unloading (HL) model	Aute (3-day) simulated microgravity induces endothelial-dependent vascular hyporesponsiveness in aortic rings via upregulation of the NO/cGMP pathway. NOS-3 expression does not change, but phosphorylation increases at activating site (Ser1177) and decreases at inhibitory site (Ser495), enhancing NOS-3 activity.	NOS-3 (eNOS), phospho-NOS-3 (Ser1177, Ser495), HSP90, NO, cGMP
[15]	Liu et al., 2022	Tail-suspension was conducted to simulate microgravity in rats	Microgravity induces myocardial atrophy and decreases cardiac function; transcriptomic and metabolomic profiling revealed significant molecular pathway alterations.	FoxO signaling, Mki67, Cdk1, Plk1, Ccna2, Cdc20, Top2a, Bub1, Ndc80, Ccnb2, Ttk; ADP ↓ (downregulated), L-glutamate ↑ (upregulated)
[16]	Liu et al., 2015	Tail-suspension model in rats	Physical inactivity (tail-suspension) increases autophagic activity, leading to cardiac dysfunction and atrophy without inducing apoptosis. Autophagy inhibition (chloroquine) reverses myocardial atrophy and restores systolic function.	Autophagy pathway (LC3, p62, Beclin-1, Vps34, mTOR)
[17]	Li et al., 2022	Human umbilical vein endothelial cells (HUVECs) exposed to 2-D clinostat-simulated microgravity in vitro	Simulated microgravity increased endothelial hyperpermeability and migration; PINK1-dependent mitophagy activation attenuated these effects	PINK1, Parkin, p62, Drp1, Mfn2, NLRP3 inflammasome,
[18]	Jiang et al., 2020	Human umbilical vein endothelial cells (HUVECs) exposed to 2D clinorotation (72 h)	Simulated microgravity induces ER stress, which activates the iNOS/NO pathway. This promotes NF-κB activation and NLRP3 inflammasome assembly, leading to increased production of pro-inflammatory cytokines (IL-1β, IL-6, TNF-α, IL-8) and endothelial apoptosis. Pharmacologic inhibition of ER stress, iNOS, NF-κB, or NLRP3 reduces these effects.	CHOP and GRP78 (ER stress markers), iNOS and NO, NF-κB/IκB signaling, NLRP3 inflammasome, and pro-inflammatory cytokines (IL-6, TNF-α, IL-8, IL-1β).
[19]	Shibata et al., 2023	13 astronauts (MRI pre/post ~155 days aboard ISS; exercise countermeasures)	Despite reduced total cardiac work in microgravity, exercise countermeasures on the ISS preserved left and right ventricular mass and function. No significant reduction in LV or RV mass postflight; modest trends toward increased LV stroke volume and ejection fraction.	-
[20]	Wang et al., 2020	Male rats under hindlimb unloading (HU) for 6 weeks with ad libitum or time-restricted feeding (TRF; 8 h/day)	Simulated microgravity (HU) caused LV dyssynchrony, reduced cardiac function, decreased PDH activity, and impaired glucose utilization. TRF preserved cardiac function and metabolism, enhanced cardiomyocyte contractility, and improved FGF21 signaling. Liver or cardiac FGF21/FGFR1 knockdown abolished TRF benefits.	FGF21, FGFR1, PDH
[21]	Sun et al., 2019	Male rats in hindlimb unloading (HU) model for 8 weeks with/without Panax quinquefolium saponin (PQS) treatment	Simulated microgravity (HU) caused cardiac remodeling, impaired function, elevated serum cardiac injury markers, and increased cardiomyocyte apoptosis. PQS treatment reduced injury markers, improved cardiac structure and function, and decreased apoptosis via AMPK activation and inhibition of Erk1/2 and CaMKII/HDAC4 pathways.	AMPK, Erk1/2, CaMKII, HDAC4, CK-MB, cTnT, IMA,
[22]	Pan et al., 2025	Mouse tail suspension and rhesus monkey bedrest models; cardiac-specific transgenic (TG) mice and AAV9-mediated overexpression	Simulated microgravity caused cardiac remodeling (fibrosis, smaller cardiomyocytes, reduced ejection fraction) and downregulated miR-199a-3p. Cardiac-specific overexpression of miR-199a-3p (transgenic mice or AAV9 delivery) mitigated remodeling and dysfunction by targeting and inhibiting MEF2C.	miR-199a-3p, MEF2C

**Table 2:** Cytoskeletal proteins and cellular function.

Protein	Function	Reference (PMID)
adducin	Endothelial barrier stabilization	[30,31]
spectrin	Cell–matrix contact and migration	[32]
MARCKS/MLP1	Adaptor protein between ion channels and membrane lipids	[33,34,35,36,37]
Ezrin	Linker protein between membrane proteins and actin cytoskeleton; migration; angiogenesis	[38]

## Abbreviations

The following abbreviations are used in this manuscript:

ACh	Acetylcholine
MARCKS	myristoylated alanine-rich C kinase substrate
MLP-1	MARCKS-like Protein-1
HU	Hindlimb unweighting
HDT	Head down tilt
HSCs	Hematopoietic stem cells
IJV	Internal jugular vein
IST	International space station
MCA	Middle cerebral arteries
MSA	Mesenteric small arteries
TS	Tail suspended
mRNA	Messenger RNA
NE	Norepinephrine
NK	Natural killer cells
NO	Nitric oxide
NOS	Nitric oxide synthase
KCl	Potassium chloride
PRI	Platelet reactivity index
SATB2	Special AT rich sequence binding protein 2
VGCC	Voltage-gated calcium channels
VWF	Von Willibrand factor
